# Crystal structure and Hirshfeld surface analysis of ethyl (4*R*,4a*S*)-2-methyl-5,8-dioxo-6-phenyl-4a,5,6,7,7a,8-hexa­hydro-4*H*-furo[2,3-*f*]iso­indole-4-carboxyl­ate

**DOI:** 10.1107/S2056989020016801

**Published:** 2021-01-08

**Authors:** Vladimir P. Zaytsev, Lala V. Chervyakova, Elena A. Sorokina, Kirill A. Vasilyev, Sevim Türktekin Çelikesir, Mehmet Akkurt, Ajaya Bhattarai

**Affiliations:** a Department of Organic Chemistry, Peoples’ Friendship University of Russia (RUDN University), 6 Miklukho-Maklaya St., 117198 Moscow, Russian Federation; b Department of Physics, Faculty of Sciences, Erciyes University, 38039 Kayseri, Turkey; c Department of Chemistry, M.M.A.M.C (Tribhuvan University), Biratnagar, Nepal

**Keywords:** crystal structure, six-membered ring, pyrrolidine ring, furan ring, Hirshfeld analysis, IMDAV reaction

## Abstract

In the title compound, C_20_H_19_NO_5_, the central six-membered ring has a slightly distorted half-chair conformation, with puckering parameters of *Q*_T_ = 0.3387 (11) Å, θ = 49.11 (19)° and φ = 167.3 (2)°. The conformation of the fused pyrrolidine ring is that of an envelope. Mol­ecules are connected by inter­molecular C—H⋯O hydrogen bonds, C—H⋯π inter­actions and π–π stacking inter­actions [centroid-to-centroid distance = 3.9536 (11) Å, with a slippage of 2.047 Å], forming a three-dimensional network. The most important contributions to the surface contacts are from H⋯H (46.3%), O⋯H/H⋯O (31.5%) and C⋯H/H⋯C (17.3%) inter­actions, as concluded from a Hirshfeld surface analysis.

## Abstract 2

The central six-membered ring of the title compound has a slightly distorted half-chair conformation while the conformation of the fused pyrrolidine ring is that of an envelope. Mol­ecules are connected by inter­molecular C—H⋯O hydrogen bonds, C—H⋯π inter­actions and π–π stacking inter­actions, forming a three dimensional network.

## Chemical context   

This work is a continuation of Diels–Alder reaction studies on vinyl­arene systems, previously carried out for the tandem acyl­ation/[4 + 2] cyclo­addition between 3-(ar­yl)allyl­amines and maleic anhydrides or acryloyl chlorides as an example of an IMDAV (the acronym for Intra Mol­ecular Diels–Alder Vinyl­arene) reaction. An IMDAV reaction is a useful tool for the one-step synthesis of benzo­furans, indoles and benzo­thio­phenes annulated with other carbo- and heterocycles (Krishna *et al.*, 2020 ▸). Previously, our group carried out a domino-sequence reaction involving acyl­ation/IMDAV/aromatization steps, which led to the target furo- and thieno[2,3-*f*]iso­indoles (Zubkov *et al.*, 2016 ▸; Horak *et al.*, 2015 ▸, 2017 ▸; Nadirova *et al.*, 2020 ▸).
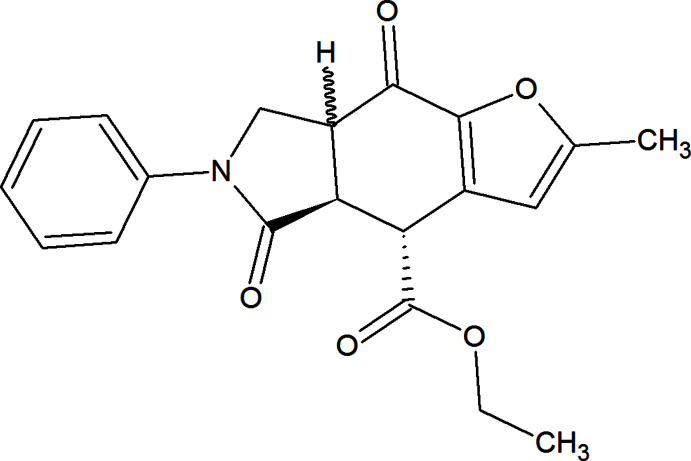


**Figure 1:**
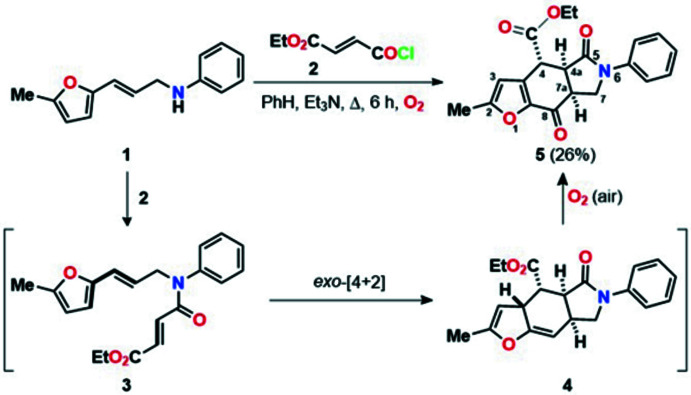
Synthesis scheme of ethyl 2-methyl-5,8-dioxo-6-phenyl-4a,5,6,7,7a,8-hexa­hydro-4*H*-furo[2,3-*f*]iso­indole-4-carboxyl­ate (**5**).

The present communication is devoted to another IMDAV reaction involving an oxidation step. We report here the first case of a three-component IMDAV/oxidation reaction between 3-(fur­yl)allyl­amine (**1**), ethyl­fumaroyl chloride (**2**) and oxygen. Unlike many other reactions, this process does not stop at the furo[2,3-*f*]iso­indole (**4**) formation but is continued by an oxidation step yielding the 8-oxofuro[2,3-*f*]iso­indole (**5**) (Fig. 1 ▸). The intra­molecular [4 + 2] cyclo­addition/oxidation sequence occurs under reflux conditions of the reaction mixture in benzene as a solvent and in ambient atmosphere; after standard purification procedures the title compound (**5**) was isolated in low yield.

Weak inter­molecular inter­actions, *e.g*. hydrogen, halogen, chalcogen, pnictogen, tetrel and triel bonding, as well as agostic, anagostic, *π–π* stacking, *n*–π*, *π*-cation and *π*-anion inter­actions, play an important role in synthesis, catalysis, crystal engineering, or mol­ecular recognition (Afkhami *et al.*, 2017 ▸; Asadov *et al.*, 2016 ▸; Gurbanov *et al.*, 2017 ▸, 2018 ▸; Karmakar *et al.*, 2017 ▸; Kopylovich *et al.*, 2011*a* ▸,*b* ▸; Ma *et al.*, 2017*a* ▸,*b* ▸; Maharramov *et al.*, 2018 ▸; Mahmoudi *et al.*, 2017 ▸, 2019 ▸; Mahmudov *et al.*, 2010 ▸, 2020 ▸; Mizar *et al.*, 2012 ▸; Sutradhar *et al.*, 2015 ▸). Herein, we highlight the role of weak inter­actions in the structural features of **5**.

## Structural commentary   

**Figure 2:**
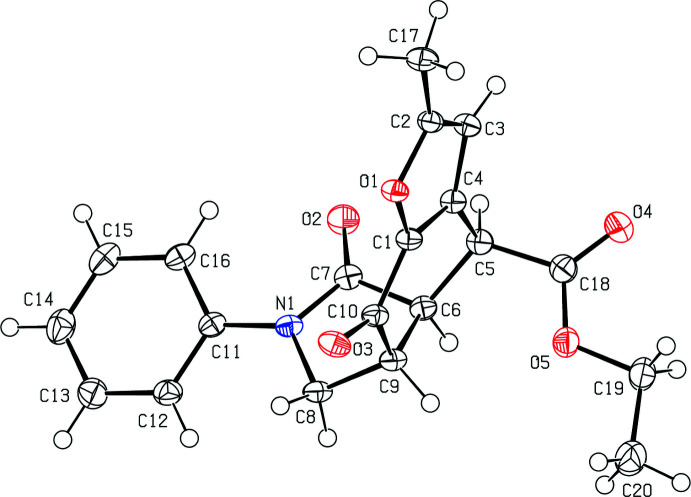
The mol­ecular structure of **5** with displacement ellipsoids for the non-hydrogen atoms drawn at the 50% probability level.

In the mol­ecule of the title compound **5** (Fig. 2 ▸), the central six-membered ring (C1/C4–C6/C9–C10) has a slightly distorted half-chair conformation, with puckering parameters (Cremer & Pople, 1975 ▸) of *Q*_T_ = 0.3387 (11) Å, θ = 49.11 (19)° and φ = 167.3 (2)°. The fused pyrrolidine ring (N1/C6–C9) adopts an envelope conformation with the C9 atom as the flap [the puckering parameters are *Q*(2) = 0.3634 (11) Å and φ(2) = 289.63 (17)°], while the fused furan ring (O1/C1–C4) is essentially planar [r.m.s. deviation = 0.001 Å]. All bond lengths and angles in the title compound (**5**) are comparable to the closely related compound (3a*R*,4*R*,4a*S*,9a*R*)-4-hy­droxy­perhydro­furo(2,3-*f*)indolizin-7(2*H*)-one (CSD refcode SIBJET; Švorc *et al.*, 2007 ▸).

## Supra­molecular features   

**Figure 3:**
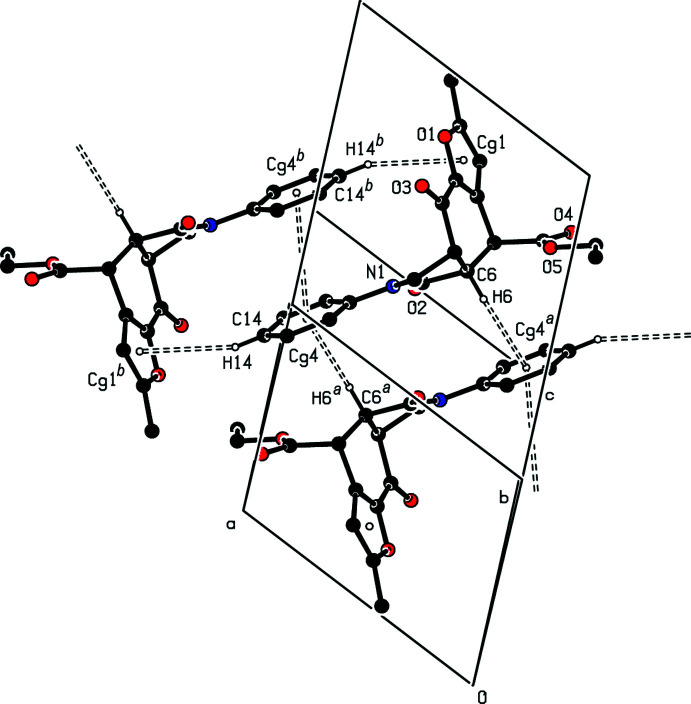
A view of the C—H⋯π inter­actions and π–π stacking inter­actions in the crystal structure of **5**. [Symmetry codes: (*a*) 1 − *x*, 1 − *y*, 1 − *z*; (*b*) 2 − *x*, 1 − *y*, 1 − *z*.]

In the crystal structure of **5**, mol­ecules are linked by two kinds of C—H⋯π inter­actions (Table 1 ▸). The first one is between an aromatic H atom (H14) of the phenyl group (C11–C16) and the centroid of the O1/C1–C4 furan ring (*Cg*1) of an adjacent mol­ecule, and the second one is between the methine H atom (H6) of the fused pyrrolidine ring (N1/C6–C9) and the centroid of the C11–C16 phenyl ring (*Cg*4) of another adjacent mol­ecule (Fig. 3 ▸).

In addition, there is a π–π stacking inter­action [*Cg*4⋯*Cg*4^i^ = 3.9536 (11) Å; symmetry code: (i) 2 − *x*, 1 − *y*, 1 − *z*], with a rather large slippage of 2.047 Å (Fig. 3 ▸).

**Figure 4:**
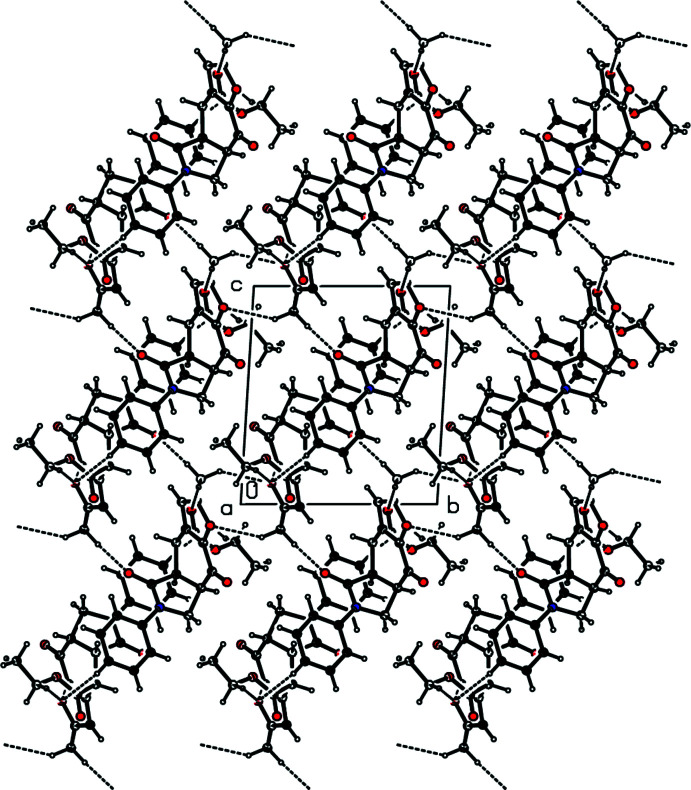
A view of the inter­molecular C—H⋯O inter­actions in the crystal structure of **5**.

The final three-dimensional network structure is completed by C—H⋯O hydrogen bonding (Fig. 4 ▸) between a phenyl H atom and the furan O atom (C14—H14⋯O1^i^), between a methyl H atom and the carbonyl O atom (C17—H17*A*⋯O4^ii^ and C17—H17*B*⋯O2^iii^), and between a methyl H atom and a methyl­ene H atom and the furan O atom (C17—H17*C*⋯O1^iv^ and C19—H19*B*⋯O1^v^). Numerical details of the hydrogen-bonding inter­actions as well as symmetry codes are given in Table 1 ▸.

## Hirshfeld surface analysis   

**Figure 5:**
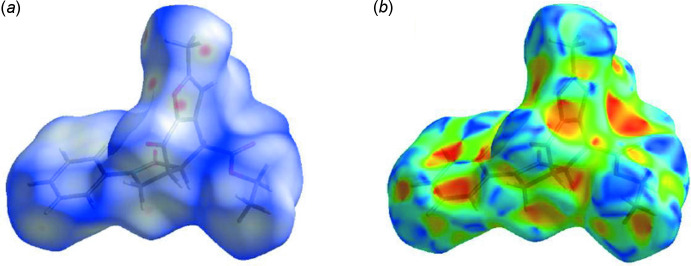
(*a*) A view of the three-dimensional Hirshfeld surface for **5**, plotted over *d*_norm_ in the range −0.2980 to 1.4527 a.u.; (*b*) Hirshfeld surface of the title compound **5** plotted over shape-index.

Hirshfeld surfaces and their associated two-dimensional fingerprint plots (McKinnon *et al.*, 2007 ▸) were used to qu­antify the various inter­molecular inter­actions, and were generated using *CrystalExplorer17* (Turner *et al.*, 2017 ▸). The shorter and longer contacts are indicated as red and blue spots on the Hirshfeld surfaces, and contacts with distances equal to the sum of the van der Waals radii are represented as white spots. Hirshfeld surfaces of the title compound **5** mapped over the normalized distance, *d*_norm_, using a standard surface resolution with a fixed colour scale of −0.2980 (red) to 1.4527 a.u. (blue) are illustrated in Fig. 5 ▸ *a*. The shape-index of the Hirshfeld surface is a tool for visualizing the π–π stacking by the presence of adjacent red and blue triangles. The plot of the Hirshfeld surface mapped over shape-index shown in Fig. 5 ▸ *b* clearly suggests that π–π inter­actions in (**5**) are significant.

**Figure 6:**
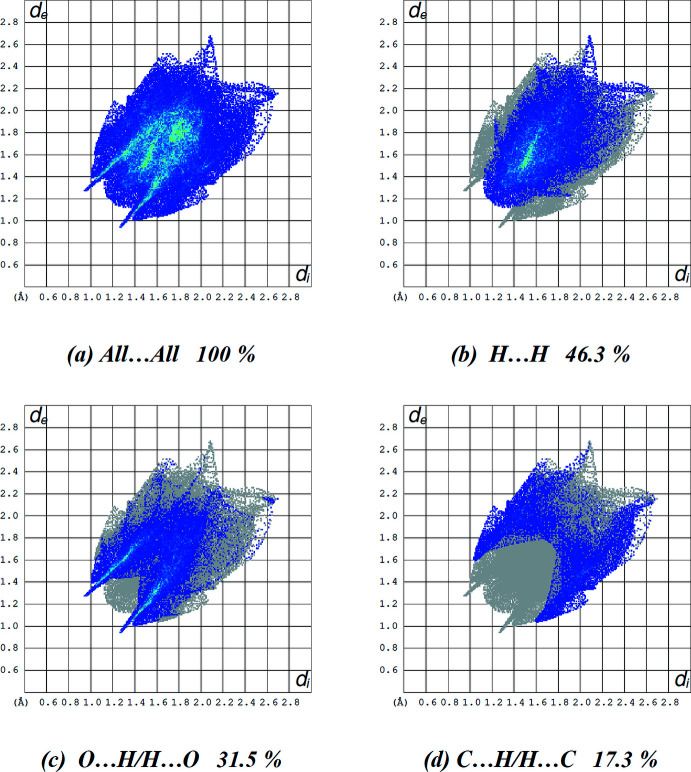
A view of the two-dimensional fingerprint plots for **5**, showing (*a*) all inter­actions, and delineated into (*b*) H⋯H, (*c*) O⋯H/H⋯O and (*d*) C⋯H/H⋯C inter­actions. The *d*_i_ and *d*_e_ values are the closest inter­nal and external distances (in Å) from given points on the Hirshfeld surface contacts.

Various inter­molecular contacts are collated in Table 2 ▸. Associated two-dimensional fingerprint plots together with their percentage contributions are shown in Fig. 6 ▸. The crystal packing is dominated by H⋯H contacts, representing van der Waals inter­actions (46.3% contribution to the overall surface), followed by O⋯H/H⋯O and C⋯H/H⋯C inter­actions, which contribute 31.5% and 17.3%, respectively. All other contacts have a minor contribution to the crystal packing.

## Database survey   

A search of the Cambridge Crystallographic Database (CSD version 5.40, update of September 2019; Groom *et al.*, 2016 ▸) yielded five entries closely related to **5**, *viz*. 2,4,6-triphenyl-7a,8-di­hydro-4*H*-furo[2,3-*f*]iso­indole-5,7(4a*H*,6*H*)-dione (CSD refcode JOGYIP; Zhou *et al.*, 2014 ▸), (4R*,4aR*,7aS*)-5-oxo-6-phenyl-4a,5,6,7,7a,8-hexa­hydro-4*H*-furo[2,3-*f*]iso­indole-4-carb­oxy­lic acid (LESXIS; Horak *et al.*, 2013 ▸), 6-benzyl-2,4,4a-trimethyl-5-oxo-4a,5,6,7,7a,8-hexa­hydro-4*H*-furo[2,3-*f*]iso­indole-4-carb­oxy­lic acid (QAFSUO; Zubkov *et al.*, 2016 ▸), 6-benzyl-4-methyl-5-oxo-4a,5,6,7,7a,8-hexa­hydro-4*H*-furo[2,3-*f*]iso­indole-4-carb­oxy­lic acid (QAFTAV; Zubkov *et al.*, 2016 ▸) and 6-allyl-5-oxo-4a,5,6,7,7a,8-hexa­hydro-4*H*-furo[2,3-*f*]iso­indole-4-carb­oxy­lic acid (QUKPAP; Horak *et al.*, 2015 ▸).

In the crystal structure of JOGYIP (space group *P* 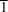), the packing is stabilized by C—H⋯O inter­molecular contacts, C—H⋯π inter­actions and π–π stacking inter­actions, forming a three-dimensional network.

In the crystal structure of LESXIS (*Pbca*), the asymmetric unit contains two mol­ecules with similar bond lengths and angles. In both mol­ecules, the conformation of the cyclo­hexene ring is that of a half-chair, while the pyrrolidinone ring adopts an envelope conformation with the γ-carbon atom of the α-pyrrolidinone ring as the flap. In the crystal, O—H⋯O hydrogen bonds between the carb­oxy­lic and carbonyl groups link alternate independent mol­ecules into chains propagating parallel to the *b-*axis direction. The crystal packing also features weak C—H⋯π inter­actions.

In the crystal structures of QAFSUO (*P*2_1_/*c*) and QAFTAV (*P*2_1_/*n*), the three-dimensional packings are stabilized by O—H⋯O inter­molecular bonds, C—H⋯O inter­molecular contacts and C—H⋯π inter­actions.

The asymmetric unit of QUKPAP (*P*2_1_/*c*) comprises two similar mol­ecules, *A* and *B*, of the same chirality. The only considerable difference concerns the conformation of the allyl group. The five-membered iso­indole rings adopt envelope conformations, whereas the six-membered rings are half-chair-puckered. The carboxyl hydrogen atoms are involved in strong hydrogen-bond formation with the carbonyl atoms of neighboring mol­ecules, giving rise to (*A*⋯*B*⋯)_*n*_ chains.

In the five structures, the different groups bonded to the central twelve-membered ring systems account for the distinct inter­molecular inter­actions in the crystals.

## Synthesis and crystallization   

Ethyl 2-methyl-5,8-dioxo-6-phenyl-4a,5,6,7,7a,8-hexa­hydro-4*H*-furo[2,3-*f*]iso­indole-4-carboxyl­ate (**5**) was synthesized according to a previously reported method (Zubkov *et al.*, 2016 ▸; Nadirova *et al.*, 2020 ▸): A solution of ethyl fumaroyl chloride (**2**; 3.6 g, 22.5 mmol) in benzene (25 ml) was added dropwise to a mixture of *N*-[(2*E*)-3-(5-methyl­furan-2-yl)prop-2-en-1-yl]aniline (**1**; 3.2 g, 15.0 mmol) with tri­ethyl­amine (4.2 ml, 30 mmol) in benzene (25 ml). The mixture was heated under reflux for 6 h. The mixture was then cooled to r.t. and poured into water (200 ml). The organic layer was separated, the aqueous layer was extracted with AcOEt (3 × 50 ml). The organic layers were combined and dried over anhydrous MgSO_4_. The extract was evaporated under reduced pressure, and the residue was crystallized at 279 K within a few days. The resulting light-beige crystals were filtered off and washed with diethyl ether (3 × 10 ml). Yield 1.4 g (26%). M.p. = 437–439 K. IR (KBr), ν (cm^−1^): 1736, 1704, 1665. ^1^H NMR (CDCl_3_, 600.2 MHz, 301 K): δ = 7.55 (*dd*, 2H, HAr, *J* = 7.6, *J* = 2.0), 7.34 (*td*, 2H, HAr, *J* = 7.6, *J* = 2.0), 7.14 (*td*, 1H, HAr, *J* = 7.6, *J* = 2.0), 6.32 (*s*, 1H, H3), 4.56 (*s*, 1H, H4), 4.43 (*dd*, 1H, H-7a, *J* = 8.5, *J* = 2.0), 4.27–4.17 (*m*, 2H, OCH_2_), 4.05 (*ddd*, 1H, H-7B, *J* = 2.0, *J* = 6.5), 3.76 (*dd*, 1H, H-4a, *J* = 1.7, *J* = 8.5), 3.51 (*td*, 1H, H-7A, *J* = 2.0, *J* = 6.5), 2.39 (*d*, 3H, CH_3_, *J* = 1.5), 1.30 (*td*, 3H, CH_2_CH_3_, *J* = 2.2, *J* = 7.2). ^13^C NMR (CDCl_3_, 150.9 MHz, 301 K): δ = 181.4, 170.9, 170.5 (CO, CO_2_, NCO), 160.9, 145.3, 138.7, 136.9, 128.8 (2C), 124.9, 119.8 (2C), 109.8, 62.0, 49.4, 45.4, 41.8, 38.7, 14.1 (CH_3_), 14.0 (CH_3_). MS (APCI): *m*/*z* = 354 [*M* + H]^+^.

## Refinement   

Crystal data, data collection and structure refinement details are summarized in Table 3 ▸. H atoms bound to C atoms were placed in geometrically idealized positions and constrained to ride on their parent atoms, with C—H = 0.95–1.00 Å and *U*_iso_(H) = 1.2 or 1.5*U*_eq_(C).

## Supplementary Material

Crystal structure: contains datablock(s) I. DOI: 10.1107/S2056989020016801/wm5592sup1.cif

Structure factors: contains datablock(s) I. DOI: 10.1107/S2056989020016801/wm5592Isup2.hkl

Supporting information file. DOI: 10.1107/S2056989020016801/wm5592Isup3.cml

CCDC reference: 2053210

Additional supporting information:  crystallographic information; 3D view; checkCIF report

## Figures and Tables

**Table 1:** Hydrogen-bond geometry (Å, °) *Cg*1 and *Cg*4 are the centroids of the furan (O1/C1–C4) and phenyl (C11–C16) rings, respectively.

*D*—H⋯*A*	*D*—H	H⋯*A*	*D*⋯*A*	*D*—H⋯*A*
C14—H14⋯O1^i^	0.95	2.53	3.4766 (16)	172
C17—H17*A*⋯O4^ii^	0.98	2.51	3.4913 (18)	176
C17—H17*B*⋯O2^iii^	0.98	2.31	3.2441 (15)	159
C17—H17*C*⋯O1^iv^	0.98	2.57	3.4232 (14)	145
C19—H19*B*⋯O1^v^	0.99	2.54	3.5181 (17)	168
C6—H6⋯*Cg*4^vi^	1.00	2.71	3.5892 (14)	146
C14—H14⋯*Cg*1^i^	0.95	2.93	3.8320 (16)	159

Symmetry codes: (i) 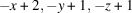; (ii) 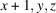; (iii) 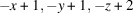; (iv) 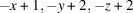; (v) 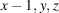; (vi) 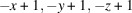.

**Table 2:** Summary of short inter­atomic contacts (Å) in the title compound **5**

Contact	Distance	Symmetry operation
H17*A*⋯O4	2.51	1 + *x*, *y*, *z*;
H14⋯O1	2.53	2 − *x*, 1 − *y*, 1 − *z*;
H17*C*⋯O1	2.57	1 − *x*, 2 − *y*, 2 − *z*;
H17*B*⋯O2	2.31	1 − *x*, 1 − *y*, 2 − *z*;
H8*A*⋯O3	2.69	1 − *x*, 2 − *y*, 1 − *z*;
O4⋯H13	2.67	−1 + *x*, *y*, 1 + *z*;
H20*C*⋯C3	3.09	−*x*, 2 − *y*, 2 − *z*;
H6⋯C16	2.72	1 − *x*, 1 − *y*, 1 − *z*;
H17*B*⋯H12	2.47	*x*, *y*, 1 + *z*;
H20*C*⋯H16	2.56	−1 + *x*, 1 + *y*, *z*.

**Table 3:** Experimental details

Crystal data	
Chemical formula	C_20_H_19_NO_5_
*M*_r_	353.36
Crystal system, space group	Triclinic, *P* 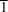
Temperature (K)	120
*a*, *b*, *c* (Å)	8.8100 (18), 9.9182 (16), 11.165 (2)
α, β, γ (°)	81.205 (7), 70.657 (6), 72.642 (4)
*V* (Å^3^)	877.0 (3)
*Z*	2
Radiation type	Mo *K*α
μ (mm^−1^)	0.10
Crystal size (mm)	0.2 × 0.2 × 0.2

Data collection	
Diffractometer	Bruker APEXII CCD
Absorption correction	Multi-scan (*SADABS*; Krause *et al.*, 2015 ▸)
*T*_min_, *T*_max_	0.659, 0.746
No. of measured, independent and observed [*I* > 2σ(*I*)] reflections	19857, 5332, 4669
*R*_int_	0.019
(sin θ/λ)_max_ (Å^−1^)	0.716

Refinement	
*R*[*F* ^2^ > 2σ(*F* ^2^)], *wR*(*F* ^2^), *S*	0.044, 0.125, 1.04
No. of reflections	5332
No. of parameters	237
H-atom treatment	H-atom parameters constrained
Δρ_max_, Δρ_min_ (e Å^−3^)	0.53, −0.18

Computer programs: *APEX2* and *SAINT* (Bruker, 2014 ▸), *SHELXT* (Sheldrick, 2015*a* ▸), *SHELXL* (Sheldrick, 2015*b* ▸), *ORTEP-3 for Windows* (Farrugia, 2012 ▸) and *PLATON* (Spek, 2020 ▸).

## supplementary crystallographic information

### Crystal data

**Table:**

C_20_H_19_NO_5_	*Z* = 2
*M**_r_* = 353.36	*F*(000) = 372
Triclinic, *P*1	*D*_x_ = 1.338 Mg m^−^^3^
*a* = 8.8100 (18) Å	Mo *K*α radiation, λ = 0.71073 Å
*b* = 9.9182 (16) Å	Cell parameters from 9941 reflections
*c* = 11.165 (2) Å	θ = 2.6–30.6°
α = 81.205 (7)°	µ = 0.10 mm^−^^1^
β = 70.657 (6)°	*T* = 120 K
γ = 72.642 (4)°	Prism, light beige
*V* = 877.0 (3) Å^3^	0.2 × 0.2 × 0.2 mm

### Data collection

**Table:**

Bruker APEXII CCD diffractometer	5332 independent reflections
Radiation source: sealed tube	4669 reflections with *I* > 2σ(*I*)
Graphite monochromator	*R*_int_ = 0.019
ω and φ scans	θ_max_ = 30.6°, θ_min_ = 2.2°
Absorption correction: multi-scan (*SADABS*; Krause *et al.*, 2015)	*h* = −12→12
*T*_min_ = 0.659, *T*_max_ = 0.746	*k* = −14→14
19857 measured reflections	*l* = −15→15

### Refinement

**Table:**

Refinement on *F*^2^	0 restraints
Least-squares matrix: full	Hydrogen site location: inferred from neighbouring sites
*R*[*F*^2^ > 2σ(*F*^2^)] = 0.044	H-atom parameters constrained
*wR*(*F*^2^) = 0.125	*w* = 1/[σ^2^(*F*_o_^2^) + (0.0747*P*)^2^ + 0.2272*P*] where *P* = (*F*_o_^2^ + 2*F*_c_^2^)/3
*S* = 1.04	(Δ/σ)_max_ = 0.001
5332 reflections	Δρ_max_ = 0.53 e Å^−^^3^
237 parameters	Δρ_min_ = −0.18 e Å^−^^3^

### Special details

**Table:**

Geometry. All esds (except the esd in the dihedral angle between two l.s. planes) are estimated using the full covariance matrix. The cell esds are taken into account individually in the estimation of esds in distances, angles and torsion angles; correlations between esds in cell parameters are only used when they are defined by crystal symmetry. An approximate (isotropic) treatment of cell esds is used for estimating esds involving l.s. planes.

### Fractional atomic coordinates and isotropic or equivalent isotropic displacement parameters (Å^2^)

**Table:**

	*x*	*y*	*z*	*U*_iso_*/*U*_eq_	
C1	0.49262 (11)	0.82060 (9)	0.82393 (8)	0.01800 (17)	
C2	0.52219 (12)	0.77526 (10)	1.01557 (9)	0.01914 (18)	
C3	0.41740 (12)	0.69939 (10)	1.01089 (9)	0.02056 (18)	
H3	0.367401	0.639266	1.077116	0.025*	
C4	0.39835 (11)	0.72876 (9)	0.88651 (9)	0.01857 (17)	
C5	0.29588 (12)	0.68264 (10)	0.82451 (9)	0.02036 (18)	
H5	0.297539	0.581687	0.852768	0.024*	
C6	0.37337 (12)	0.69298 (10)	0.67917 (9)	0.01928 (17)	
H6	0.289113	0.686876	0.639739	0.023*	
C7	0.52879 (12)	0.57073 (10)	0.63456 (9)	0.02000 (18)	
C8	0.56409 (13)	0.76769 (10)	0.49121 (9)	0.02031 (18)	
H8A	0.508910	0.774189	0.425593	0.024*	
H8B	0.650611	0.820227	0.458834	0.024*	
C9	0.43715 (12)	0.82351 (9)	0.61653 (9)	0.01886 (17)	
H9	0.343898	0.902634	0.599506	0.023*	
C10	0.52459 (12)	0.87442 (9)	0.69242 (9)	0.01830 (17)	
C11	0.78337 (12)	0.53580 (10)	0.44672 (9)	0.01931 (18)	
C12	0.84407 (13)	0.58679 (11)	0.32111 (10)	0.0241 (2)	
H12	0.786842	0.676487	0.292163	0.029*	
C13	0.98773 (14)	0.50692 (13)	0.23836 (11)	0.0308 (2)	
H13	1.028278	0.541994	0.153099	0.037*	
C14	1.07198 (14)	0.37572 (13)	0.28046 (12)	0.0321 (2)	
H14	1.169324	0.320347	0.223866	0.039*	
C15	1.01305 (14)	0.32603 (11)	0.40575 (11)	0.0283 (2)	
H15	1.071538	0.236753	0.434436	0.034*	
C16	0.87003 (13)	0.40465 (10)	0.48986 (10)	0.02336 (19)	
H16	0.831446	0.369962	0.575521	0.028*	
C17	0.59178 (14)	0.78967 (11)	1.11582 (10)	0.0247 (2)	
H17A	0.711689	0.780661	1.078853	0.037*	
H17B	0.573698	0.715273	1.183471	0.037*	
H17C	0.535888	0.882592	1.151301	0.037*	
C18	0.11631 (12)	0.77419 (11)	0.87182 (10)	0.02347 (19)	
C19	−0.08020 (14)	0.98413 (12)	0.82766 (12)	0.0312 (2)	
H19A	−0.098723	1.029690	0.906094	0.037*	
H19B	−0.166938	0.933475	0.843495	0.037*	
C20	−0.08800 (16)	1.09358 (14)	0.71871 (14)	0.0416 (3)	
H20A	−0.064035	1.046628	0.640874	0.062*	
H20B	−0.005259	1.146023	0.706811	0.062*	
H20C	−0.199768	1.159291	0.737594	0.062*	
N1	0.63587 (10)	0.61915 (8)	0.52807 (8)	0.01870 (16)	
O1	0.56926 (8)	0.85082 (7)	0.90205 (6)	0.01874 (14)	
O2	0.55020 (10)	0.45051 (8)	0.68426 (8)	0.02855 (17)	
O3	0.61486 (10)	0.95404 (8)	0.64330 (7)	0.02436 (16)	
O4	0.01858 (11)	0.75061 (11)	0.97188 (8)	0.0372 (2)	
O5	0.08563 (9)	0.88584 (8)	0.79139 (8)	0.02685 (17)	

### Atomic displacement parameters (Å^2^)

**Table:**

	*U*^11^	*U*^22^	*U*^33^	*U*^12^	*U*^13^	*U*^23^
C1	0.0213 (4)	0.0180 (4)	0.0169 (4)	−0.0062 (3)	−0.0084 (3)	0.0004 (3)
C2	0.0218 (4)	0.0193 (4)	0.0156 (4)	−0.0045 (3)	−0.0065 (3)	0.0010 (3)
C3	0.0240 (4)	0.0211 (4)	0.0172 (4)	−0.0079 (3)	−0.0070 (3)	0.0024 (3)
C4	0.0204 (4)	0.0182 (4)	0.0183 (4)	−0.0059 (3)	−0.0071 (3)	−0.0001 (3)
C5	0.0216 (4)	0.0207 (4)	0.0210 (4)	−0.0082 (3)	−0.0077 (3)	0.0001 (3)
C6	0.0212 (4)	0.0191 (4)	0.0201 (4)	−0.0062 (3)	−0.0088 (3)	−0.0009 (3)
C7	0.0228 (4)	0.0182 (4)	0.0220 (4)	−0.0068 (3)	−0.0095 (3)	−0.0008 (3)
C8	0.0277 (4)	0.0163 (4)	0.0171 (4)	−0.0030 (3)	−0.0101 (3)	0.0005 (3)
C9	0.0232 (4)	0.0170 (4)	0.0176 (4)	−0.0040 (3)	−0.0093 (3)	0.0000 (3)
C10	0.0229 (4)	0.0151 (4)	0.0170 (4)	−0.0041 (3)	−0.0075 (3)	0.0000 (3)
C11	0.0215 (4)	0.0192 (4)	0.0208 (4)	−0.0050 (3)	−0.0105 (3)	−0.0031 (3)
C12	0.0258 (5)	0.0264 (5)	0.0212 (4)	−0.0049 (4)	−0.0105 (4)	−0.0015 (3)
C13	0.0279 (5)	0.0398 (6)	0.0237 (5)	−0.0050 (4)	−0.0082 (4)	−0.0059 (4)
C14	0.0254 (5)	0.0362 (6)	0.0333 (6)	−0.0004 (4)	−0.0091 (4)	−0.0128 (5)
C15	0.0256 (5)	0.0238 (5)	0.0377 (6)	−0.0007 (4)	−0.0156 (4)	−0.0073 (4)
C16	0.0258 (5)	0.0203 (4)	0.0273 (5)	−0.0041 (3)	−0.0140 (4)	−0.0019 (3)
C17	0.0318 (5)	0.0269 (5)	0.0200 (4)	−0.0102 (4)	−0.0133 (4)	0.0021 (3)
C18	0.0220 (4)	0.0282 (5)	0.0236 (4)	−0.0104 (4)	−0.0081 (3)	−0.0012 (4)
C19	0.0214 (5)	0.0308 (5)	0.0354 (6)	−0.0035 (4)	−0.0043 (4)	−0.0008 (4)
C20	0.0310 (6)	0.0337 (6)	0.0474 (7)	−0.0021 (5)	−0.0057 (5)	0.0073 (5)
N1	0.0232 (4)	0.0154 (3)	0.0183 (3)	−0.0037 (3)	−0.0092 (3)	0.0003 (3)
O1	0.0228 (3)	0.0200 (3)	0.0160 (3)	−0.0080 (2)	−0.0081 (2)	0.0012 (2)
O2	0.0321 (4)	0.0176 (3)	0.0336 (4)	−0.0080 (3)	−0.0081 (3)	0.0041 (3)
O3	0.0334 (4)	0.0215 (3)	0.0213 (3)	−0.0130 (3)	−0.0090 (3)	0.0028 (3)
O4	0.0260 (4)	0.0512 (5)	0.0276 (4)	−0.0097 (4)	−0.0042 (3)	0.0070 (4)
O5	0.0211 (3)	0.0244 (4)	0.0303 (4)	−0.0051 (3)	−0.0040 (3)	0.0017 (3)

### Geometric parameters (Å, º)

**Table:**

C1—C4	1.3712 (12)	C11—C12	1.3987 (14)
C1—O1	1.3775 (11)	C11—C16	1.4023 (13)
C1—C10	1.4478 (12)	C11—N1	1.4156 (12)
C2—C3	1.3712 (13)	C12—C13	1.3906 (15)
C2—O1	1.3736 (11)	C12—H12	0.9500
C2—C17	1.4849 (13)	C13—C14	1.3904 (17)
C3—C4	1.4294 (13)	C13—H13	0.9500
C3—H3	0.9500	C14—C15	1.3893 (18)
C4—C5	1.5035 (13)	C14—H14	0.9500
C5—C18	1.5305 (14)	C15—C16	1.3897 (15)
C5—C6	1.5385 (14)	C15—H15	0.9500
C5—H5	1.0000	C16—H16	0.9500
C6—C7	1.5282 (14)	C17—H17A	0.9800
C6—C9	1.5420 (13)	C17—H17B	0.9800
C6—H6	1.0000	C17—H17C	0.9800
C7—O2	1.2239 (12)	C18—O4	1.2031 (13)
C7—N1	1.3737 (12)	C18—O5	1.3356 (13)
C8—N1	1.4734 (12)	C19—O5	1.4587 (13)
C8—C9	1.5316 (13)	C19—C20	1.5066 (18)
C8—H8A	0.9900	C19—H19A	0.9900
C8—H8B	0.9900	C19—H19B	0.9900
C9—C10	1.5369 (13)	C20—H20A	0.9800
C9—H9	1.0000	C20—H20B	0.9800
C10—O3	1.2289 (12)	C20—H20C	0.9800
			
C4—C1—O1	110.30 (8)	C12—C11—N1	118.92 (8)
C4—C1—C10	128.08 (8)	C16—C11—N1	121.42 (9)
O1—C1—C10	121.47 (8)	C13—C12—C11	120.37 (10)
C3—C2—O1	110.50 (8)	C13—C12—H12	119.8
C3—C2—C17	133.13 (9)	C11—C12—H12	119.8
O1—C2—C17	116.36 (8)	C14—C13—C12	119.97 (11)
C2—C3—C4	106.32 (8)	C14—C13—H13	120.0
C2—C3—H3	126.8	C12—C13—H13	120.0
C4—C3—H3	126.8	C15—C14—C13	119.64 (10)
C1—C4—C3	106.46 (8)	C15—C14—H14	120.2
C1—C4—C5	121.28 (8)	C13—C14—H14	120.2
C3—C4—C5	132.22 (8)	C14—C15—C16	121.15 (10)
C4—C5—C18	107.45 (8)	C14—C15—H15	119.4
C4—C5—C6	109.46 (8)	C16—C15—H15	119.4
C18—C5—C6	113.92 (8)	C15—C16—C11	119.18 (10)
C4—C5—H5	108.6	C15—C16—H16	120.4
C18—C5—H5	108.6	C11—C16—H16	120.4
C6—C5—H5	108.6	C2—C17—H17A	109.5
C7—C6—C5	111.40 (8)	C2—C17—H17B	109.5
C7—C6—C9	102.38 (7)	H17A—C17—H17B	109.5
C5—C6—C9	118.72 (8)	C2—C17—H17C	109.5
C7—C6—H6	107.9	H17A—C17—H17C	109.5
C5—C6—H6	107.9	H17B—C17—H17C	109.5
C9—C6—H6	107.9	O4—C18—O5	124.84 (10)
O2—C7—N1	126.77 (9)	O4—C18—C5	123.54 (10)
O2—C7—C6	125.30 (9)	O5—C18—C5	111.55 (8)
N1—C7—C6	107.91 (8)	O5—C19—C20	106.88 (9)
N1—C8—C9	102.56 (7)	O5—C19—H19A	110.3
N1—C8—H8A	111.3	C20—C19—H19A	110.3
C9—C8—H8A	111.3	O5—C19—H19B	110.3
N1—C8—H8B	111.3	C20—C19—H19B	110.3
C9—C8—H8B	111.3	H19A—C19—H19B	108.6
H8A—C8—H8B	109.2	C19—C20—H20A	109.5
C8—C9—C10	109.49 (8)	C19—C20—H20B	109.5
C8—C9—C6	102.03 (7)	H20A—C20—H20B	109.5
C10—C9—C6	114.13 (7)	C19—C20—H20C	109.5
C8—C9—H9	110.3	H20A—C20—H20C	109.5
C10—C9—H9	110.3	H20B—C20—H20C	109.5
C6—C9—H9	110.3	C7—N1—C11	126.27 (8)
O3—C10—C1	123.57 (9)	C7—N1—C8	111.65 (8)
O3—C10—C9	121.65 (8)	C11—N1—C8	121.13 (8)
C1—C10—C9	114.77 (8)	C2—O1—C1	106.42 (7)
C12—C11—C16	119.66 (9)	C18—O5—C19	116.94 (8)

### Hydrogen-bond geometry (Å, º)

*Cg*1 and *Cg*4 are the centroids of the furan (O1/C1–C4) and phenyl (C11–C16) rings, respectively.

**Table:**

*D*—H···*A*	*D*—H	H···*A*	*D*···*A*	*D*—H···*A*
C14—H14···O1^i^	0.95	2.53	3.4766 (16)	172
C17—H17*A*···O4^ii^	0.98	2.51	3.4913 (18)	176
C17—H17*B*···O2^iii^	0.98	2.31	3.2441 (15)	159
C17—H17*C*···O1^iv^	0.98	2.57	3.4232 (14)	145
C19—H19*B*···O1^v^	0.99	2.54	3.5181 (17)	168
C6—H6···*Cg*4^vi^	1.00	2.71	3.5892 (14)	146
C14—H14···*Cg*1^i^	0.95	2.93	3.8320 (16)	159

Symmetry codes: (i) −*x*+2, −*y*+1, −*z*+1; (ii) *x*+1, *y*, *z*; (iii) −*x*+1, −*y*+1, −*z*+2; (iv) −*x*+1, −*y*+2, −*z*+2; (v) *x*−1, *y*, *z*; (vi) −*x*+1, −*y*+1, −*z*+1.
